# Oral levamisole as a route-decoupled adjunct to intramuscular foot-and-mouth disease vaccination: a two-species evaluation

**DOI:** 10.3389/fvets.2026.1918567

**Published:** 2026-09-23

**Authors:** Hyeong Won Kim, So Hui Park, Mi-Kyeong Ko, Seokwon Shin, Jong-Hyeon Park, Min Ja Lee

**Affiliations:** Center for Foot-and-Mouth Disease Vaccine Research, Animal and Plant Quarantine Agency, Gimcheon-si, Gyeongsangbuk-do, Republic of Korea

**Keywords:** foot-and-mouth disease virus, immunomodulation, mice and swine, oral levamisole, route-decoupled

## Abstract

**Introduction:**

Foot-and-mouth disease (FMD) control still depends largely on inactivated vaccines; however, improving vaccine efficacy without reformulating existing vaccines remains an important practical challenge.

**Methods:**

We assessed oral levamisole as an independently administered adjunct alongside, but separately from, a conventional intramuscular bivalent FMD vaccine in mice and FMD-seronegative swine. Serotype-specific immune responses were assessed using structural protein (SP) ELISA and virus-neutralization (VN) assays. IgA-associated responses were examined in mouse serum and saliva and in swine serum, while cytokine-related gene transcription was profiled in porcine peripheral blood mononuclear cells. Murine post-challenge outcomes and porcine safety were also evaluated.

**Results:**

Oral levamisole increased antibody and neutralizing responses against FMD virus (FMDV) serotypes O and A in both species. In mice, adjunct treatment improved post-challenge survival (5/5 [100%] with vaccine + levamisole vs. 2/5 [40%] with vaccine alone; log-rank *p* = 0.004), reduced post-challenge body weight loss, and increased IgA-associated responses.

**Discussion:**

These findings support oral levamisole as a practical route-decoupled adjunct for enhancing FMD vaccine responsiveness without requiring modification of the injectable vaccine formulation.

## 1 Introduction

Foot-and-mouth disease (FMD) remains one of the most economically devastating infections of cloven-hoofed livestock because outbreaks rapidly disrupt animal movement, productivity, and international trade. Inactivated vaccines are indispensable components of FMD control programs; however, there is continuing pressure to improve the strength and reliability of vaccine-induced protection under practical field conditions (1–7).

In veterinary vaccination programs, the central translational challenge is not simply whether immunity can be increased, but whether it can be increased without redesigning a vaccine that is already manufacturable and deployable. Changes to antigen formulations, adjuvant compositions, or emulsion properties may strengthen vaccine-induced immune responses, but they can also introduce additional manufacturing, regulatory, and stability challenges. Accordingly, separately administered adjunct strategies are attractive because they seek to support host immunity while preserving the vaccine itself as a fixed platform (8, 9).

This issue is particularly relevant for FMD virus (FMDV), as natural infection begins at upper aerodigestive mucosal surfaces, whereas conventional intramuscular vaccination is evaluated primarily through serum antibody responses. An ideal supportive strategy would therefore augment systemic immunity while extending the quality and coordination of vaccine-induced responses, especially during the early post-immunization period when protective immunity is being established (10–17).

One approach to achieving this objective is route-decoupled immunomodulation, in which the vaccine and immunomodulatory adjunct are administered independently, often through different routes and schedules, allowing adjunct dosing to be adjusted without altering the vaccine formulation (8, 9, 18).

Levamisole is well suited to testing this concept. Originally developed as an imidazothiazole anthelmintic, it has a long history of veterinary use, favorable oral bioavailability, and recognized immunomodulatory activity across multiple animal species (19, 20). Besides its antiparasitic effects, levamisole has been associated with enhanced antigen-presenting cell activity, lymphocyte stimulation, and adjuvant-like support of immune responses to inactivated viral vaccines, including FMD-related formulations (21–23).

From a pharmacological perspective, levamisole differs from nucleoside-like or antiviral immunomodulators because its reported effects are linked to cAMP-related signaling pathways, cholinergic regulation, and broader modulation of immune-cell activation states. These characteristics make it a useful model compound for determining whether a metabolically and immunopharmacologically distinct small molecule can enhance vaccine responsiveness when administered orally as a separate regimen rather than as an incorporated vaccine component (20, 21, 24).

Levamisole has previously been shown to enhance FMD vaccine-induced immunity when incorporated directly into the vaccine formulation and injected as a built-in adjuvant (22, 23). It remained unknown, however, whether this benefit is retained when levamisole is administered orally and independently of the vaccine.

Accordingly, we investigated whether levamisole could enhance vaccine responsiveness when administered orally as a regimen independent of the injected vaccine. To address this question, we employed a two-species design combining challenge-based proof-of-concept testing in mice with target-species evaluation in swine. We assessed serotype-specific antibody responses, virus-neutralizing activity, IgA-associated responses, transcriptional changes in porcine peripheral blood mononuclear cells (PBMCs), murine post-challenge outcomes, and porcine safety to determine whether separately administered levamisole could reinforce intramuscular FMD vaccination without requiring vaccine reformulation.

## 2 Materials and methods

### 2.1 Chemicals and reagents

Levamisole hydrochloride (cat. no. Y0000047) was purchased from Sigma-Aldrich (St. Louis, MO, USA) and served as the oral adjunct in this study. ISA 206 was obtained from SEPPIC (Paris, France), and Quil-A was purchased from InvivoGen (San Diego, CA, USA). Structural protein (SP) ELISAs were performed using the PrioCHECK™ FMDV Type O Antibody ELISA Kit (cat. no. 7610420) and the PrioCHECK™ FMDV Type A Antibody ELISA Kit (cat. no. 7610850), both from Prionics Lelystad B. V. (Lelystad, The Netherlands). TRIzol reagent was purchased from Thermo Fisher Scientific (Waltham, MA, USA), the RNeasy Mini Kit from QIAGEN (Valencia, CA, USA), the GoScript Reverse Transcription System from Promega (Madison, WI, USA), and iQ SYBR Green Supermix from Bio-Rad Laboratories (Hercules, CA, USA). Pilocarpine hydrochloride, used to stimulate salivation before saliva collection, was also obtained from Sigma-Aldrich.

### 2.2 FMDV antigen production

FMDV-derived antigens were prepared from the O PA2 (O/PAK/44/2008) and A YC (A/SKR/YC/2017) strains propagated in BHK-21 cell monolayers. Viral inactivation was performed using 0.003 N binary ethylenimine (BEI), followed by purification through sucrose-density-gradient ultracentrifugation as previously described (22). Complete viral inactivation was confirmed by serial passage in BHK-21 cells.

### 2.3 Experimental animals

Female C57BL/6 N mice (6–7 weeks old; *n* = 5 per group) and FMD antibody-negative swine (8–9 weeks old; *n* = 5 per group) were maintained in ABSL-3 facilities under standard husbandry conditions. Animals were monitored throughout the study in accordance with institutional animal-care guidelines. All experimental procedures were approved by the Institutional Animal Care and Use Committee (IACUC) of the Animal and Plant Quarantine Agency (IACUC Nos. APQA-2025-1608 and APQA-2026-2184). Group sizes were determined on the basis of precedent from previous FMD vaccine-adjunct studies using comparable immunological endpoints, together with feasibility constraints associated with ABSL-3 containment and large-animal experimentation.

### 2.4 Experimental design and treatment protocol

Following acclimatization, animals were randomly assigned to treatment groups before vaccination and adjunct administration. Mice (mean body weight approximately 19 g at the start of treatment; Supplementary Table 2) received 100 μg per dose (≈5.2 mg/kg) by oral gavage, whereas swine received 5 mg per dose using an oral dosing syringe. The PBS and vaccine-only control groups received the vehicle alone by the same oral route and on the same dosing schedule (daily from 0 to 28 dpv, then weekly from 28 to 56 dpv). Swine body weights were estimated from the age and breed of the animals with reference to established growth-performance data for Landrace and crossbred finishing pigs (25, 26): approximately 15–20 kg at the start of treatment (8–9 weeks of age; ≈0.25–0.33 mg/kg), increasing to approximately 60–75 kg by 56 dpv (≈0.07–0.08 mg/kg) and approaching market weight (≈110 kg) by the end of the observation period (84 dpv) (27). Swine body weights were not measured at each time point, and values are therefore presented as literature-based estimates. Clinical observation by the attending veterinarian indicated that body weight gain in the levamisole-treated group was comparable to that of the PBS control group throughout the study period. Investigators responsible for immunological assays and outcome assessments were blinded to group allocation. In mice, body weight and food intake were additionally recorded, and the food efficiency ratio (FER) was calculated as body weight gain divided by food intake (× 100); these data are provided in Supplementary Tables 2, 3. All quantitative assays were performed in technical triplicates, which were averaged per animal before analysis.

### 2.5 Vaccination protocol

A bivalent inactivated FMD vaccine containing O PA2 and A YC antigens was administered intramuscularly on day 0. In mice, each 100 μL dose contained 0.375 μg of O PA2 antigen and 0.375 μg of A YC antigen formulated with ISA 206 (50:50, w/w), Al(OH)₃ (10% per dose), and Quil-A (15 μg per dose). In swine, each 1 mL dose contained 15 μg of O PA2 antigen and 15 μg of A YC antigen formulated with ISA 206 (50:50, w/w), Al(OH)₃ (10% per dose), and Quil-A (150 μg per dose). Swine received a booster vaccination on day 28. Control animals received either PBS or vaccine alone according to group assignment.

### 2.6 Sample collection and PBMC isolation

Blood samples were collected at predetermined intervals throughout the study to monitor immune responses longitudinally. Sampling was performed at 0, 7, 14, 21, 28, 35, 42, 56, 70, and 84 dpv. PBMCs were isolated by Ficoll density-gradient centrifugation for transcriptional analyses. Saliva samples were collected following pilocarpine hydrochloride administration to assess IgA-associated responses related to mucosal immunity.

### 2.7 ELISA and virus neutralization tests

SP-specific antibody responses against FMDV serotypes O and A were measured using the PrioCHECK™ FMDV Type O Antibody ELISA Kit and the PrioCHECK™ FMDV Type A Antibody ELISA Kit, respectively, according to the manufacturer’s instructions. Functional antibody responses were evaluated by virus-neutralization (VN) assays using the FMDV O PA2 and A YC strains. All assays were performed according to established laboratory protocols (22), with live-virus procedures conducted under biosafety level 3 (BSL-3) containment at APQA in accordance with established laboratory and WOAH procedures.

### 2.8 IgA analysis

IgA-associated responses were quantified by ELISA using species- and sample-appropriate assays. In mice, sIgA was measured in serum and in saliva using a mouse sIgA-specific ELISA kit (CSB-E08413m; Cusabio, Wuhan, China), which is validated for serum, plasma, tissue homogenates, and bronchoalveolar lavage fluid; saliva was collected after pilocarpine hydrochloride administration. In swine, serum IgA was measured in serial serum samples using a porcine secretory IgA (sIgA)-specific ELISA kit (Cloud-Clone Corp., TX, USA); because the measurement was performed in serum, the results are reported as serum IgA-associated responses. Because the mouse assay specifically detects secretory-component–associated IgA, the measured mouse analyte is designated sIgA; however, serum sIgA was not interpreted as direct evidence of protective mucosal immunity, because mucosal tissues and viral shedding were not assessed.

### 2.9 Quantitative RT-PCR

Total RNA was extracted from porcine PBMCs using TRIzol reagent and further purified using the RNeasy Mini Kit. Purified RNA was reverse-transcribed into cDNA using the GoScript Reverse Transcription System. Quantitative RT-PCR was performed with gene-specific primers listed in Supplementary Table 1. Relative expression levels of immune-related genes were normalized to HPRT and calculated as fold changes relative to the appropriate comparator group (22).

### 2.10 Challenge study in mice

To assess post-vaccination protection, mice were challenged intraperitoneally with FMDV O/VET/2013 at 84 dpv using a dose equivalent to 100 LD₅₀. Survival and body weight changes were monitored for 7 days following challenge.

### 2.11 Safety assessment in swine

Safety was assessed in swine through clinical observations and serum biochemical analyses. Liver- and kidney-related parameters, including Alanine aminotransferase (ALT), Aspartate aminotransferase (AST), blood urea nitrogen (BUN), Creatinine, Lactate dehydrogenase (LDH), total protein (TP), albumin (ALB), and Albumin/Globulin (A/G) ratio, were monitored to assess the tolerability of the oral adjunct regimen.

### 2.12 Statistical analysis

Longitudinal datasets were analyzed using two-way repeated-measures ANOVA (Geisser–Greenhouse correction), with treatment and time as factors and repeated observations matched within animals; *post hoc* comparisons were performed with Tukey’s multiple-comparison test, and multiplicity-adjusted *p* values are reported. Virus-neutralizing titers were log_10_-transformed before analysis. Survival data were analyzed using the Kaplan–Meier method, and differences between groups were compared with the log-rank (Mantel–Cox) test. Cytokine-related gene transcription (single time point) was analyzed by ordinary one-way ANOVA followed by Tukey’s multiple-comparison test; for genes showing unequal variances (IFNγ, IL-18, and IL-23R), Welch’s ANOVA followed by Dunnett’s T3 multiple-comparison test was used. Exact adjusted *p* values are provided in the Supplementary Statistical Analysis file and summarized in Supplementary Table 4. Statistical significance was set at *p* < 0.05.

## 3 Results

### 3.1 Oral levamisole enhanced vaccine-induced antibody responses in mice

We first investigated whether oral levamisole administration could influence the immunogenicity of intramuscular FMD vaccination in mice. The experimental schedule is shown in Figure 1A. Compared with mice that received vaccine alone, animals treated with oral levamisole in combination with vaccination showed stronger SP-specific antibody responses against both serotype O and serotype A throughout the observation period (Figures 1B,C). A similar pattern was observed in the VN assay, in which the levamisole-treated group demonstrated higher neutralizing activity against both the O PA2 and A YC strains than the vaccine-only group (Figures 1D,E). Collectively, these findings indicated that oral levamisole administration enhanced vaccine-induced humoral responses in the murine model.

**Figure 1:**
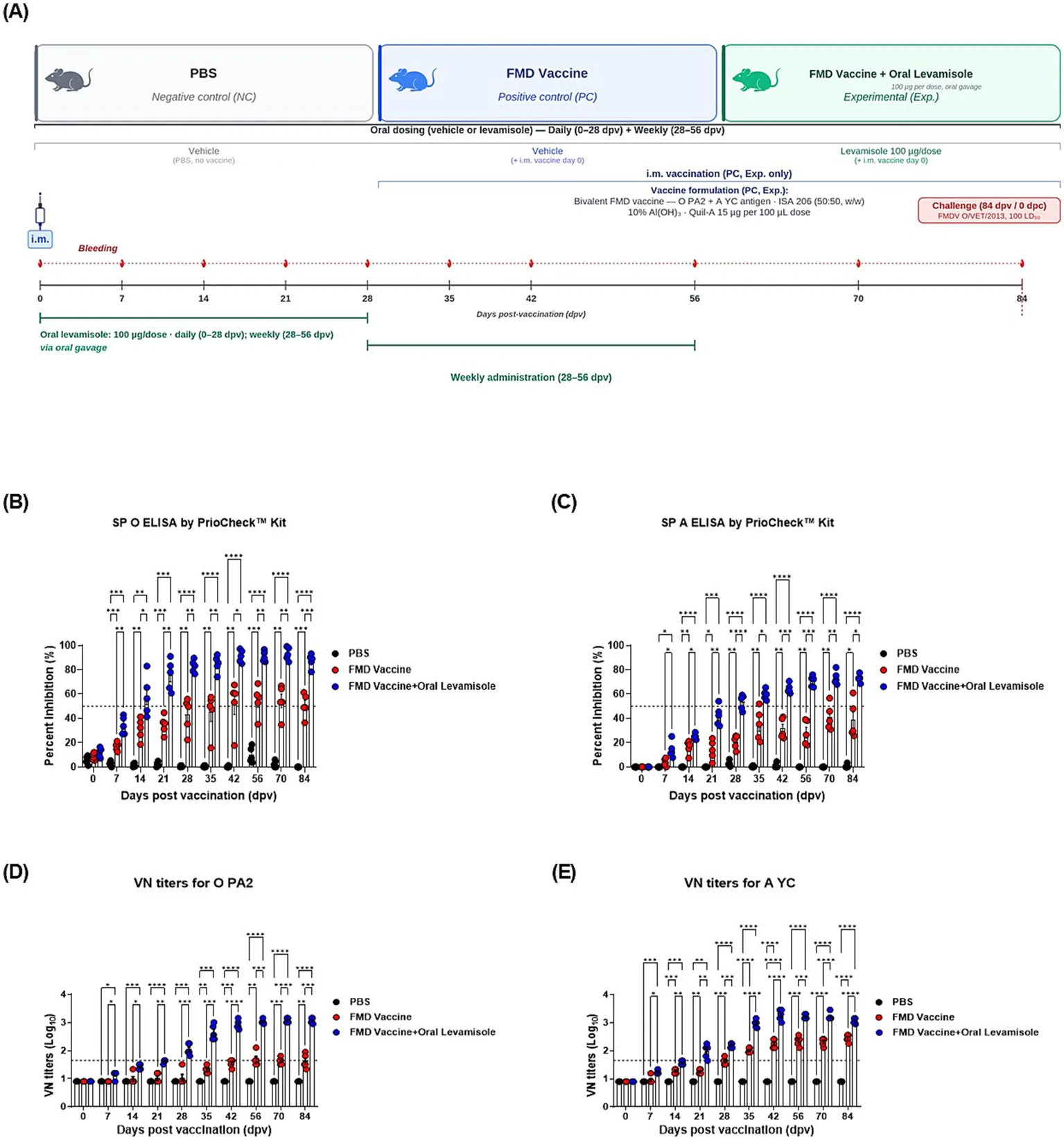
Oral levamisole administration enhanced vaccine-induced humoral responses in mice. **(A)** Schematic overview of the mouse study design. Mice were immunized intramuscularly with a bivalent inactivated FMD vaccine on day 0 and received oral levamisole according to the indicated schedule. **(B,C)** Structural protein (SP)-specific antibody responses against serotypes O and A, respectively. **(D,E)** Virus-neutralizing (VN) antibody titers against the O PA2 and A YC strains, respectively. Oral levamisole administration enhanced vaccine-induced antibody responses compared with vaccination alone. Data are presented as mean ± SEM. Statistical significance is indicated as follows: *p < 0.05, **p < 0.01, ***p < 0.001, and ****p < 0.0001. Statistical significance: two-way repeated-measures ANOVA (Geisser–Greenhouse) with Tukey-adjusted *p* values (*p < 0.05, **p < 0.01, ***p < 0.001, ****p < 0.0001).

### 3.2 Oral levamisole was associated with improved post-challenge outcomes in mice

To determine whether the immunological enhancement induced by oral levamisole was accompanied by improved protection, vaccinated mice were challenged with FMDV at 84 dpv. As shown in Figure 2A, the vaccine + oral levamisole group showed improved survival compared with the vaccine-only group: the survival proportions were 0/5 (0%; 95% CI, 0–45.1%) in the PBS group, 2/5 (40%; 95% CI, 5.3–85.3%) in the vaccine-only group, and 5/5 (100%; 95% CI, 47.8–100%) in the vaccine + levamisole group (log-rank [Mantel–Cox] test, χ^2^ = 11.04, df = 2, *p* = 0.0040). Consistent with this finding, body weight loss following challenge was less pronounced in levamisole-treated mice, and recovery was more favorable throughout the monitoring period (Figure 2B). These results suggested that the route-decoupled levamisole regimen was associated with improved post-challenge outcomes in vaccinated mice.

**Figure 2:**
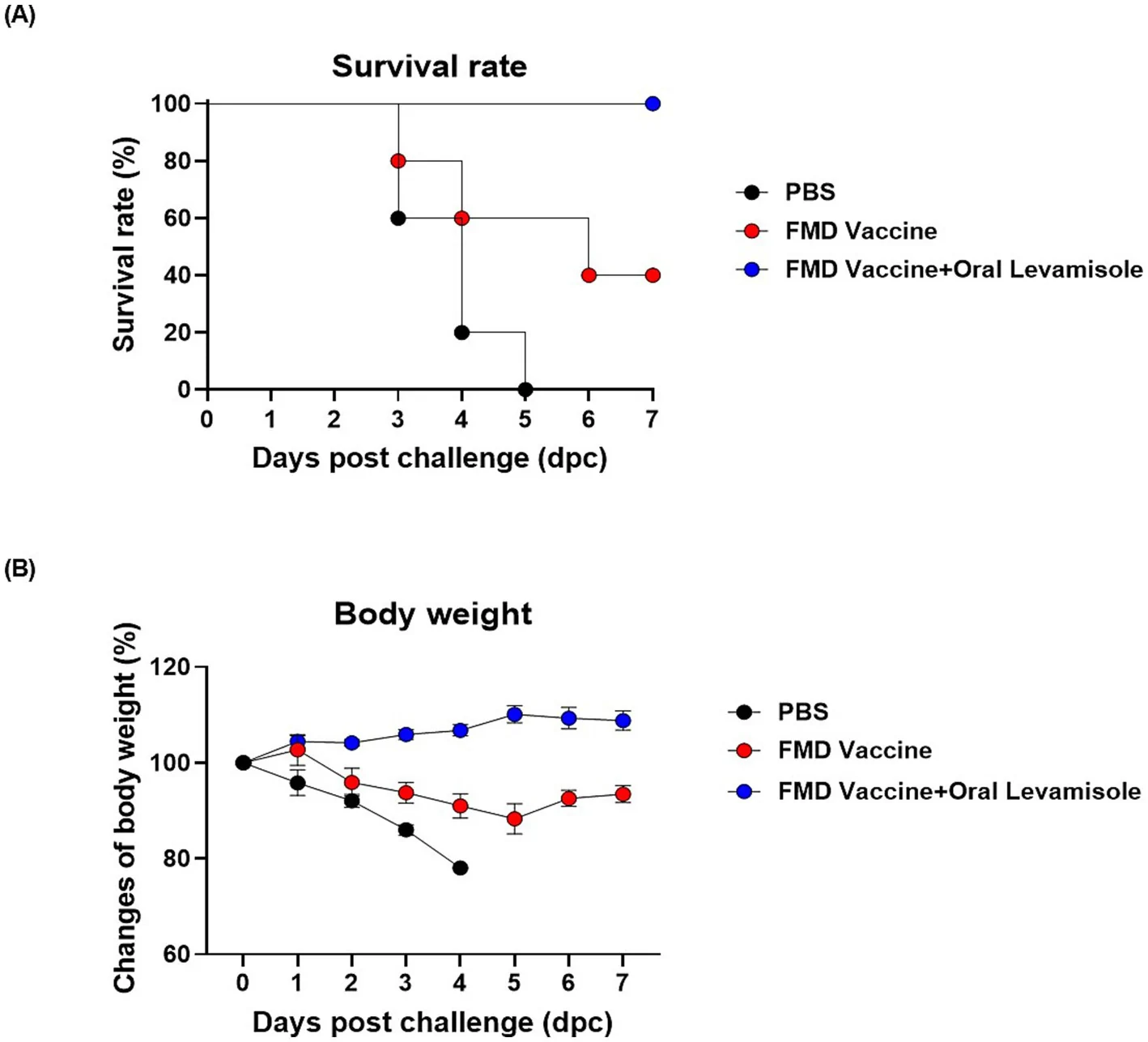
Oral levamisole administration was associated with improved post-challenge outcomes in vaccinated mice. **(A)** Survival curves of mice following challenge with FMDV O/VET/2013. **(B)** Body weight changes after challenge. Mice receiving oral levamisole in combination with intramuscular vaccination showed improved survival and reduced body weight loss compared with mice receiving vaccine alone. Survival data were analyzed using the log-rank test. Body weight data are presented as mean ± SEM (*n* = 5 per group; survivors: PBS 0/5, vaccine 2/5, vaccine + levamisole 5/5; log-rank [Mantel–Cox] *p* = 0.0040). O PA2 (vaccine) and O/VET/2013 (challenge) are distinct serotype O isolates (intratypic heterologous challenge).

### 3.3 Oral levamisole enhanced and sustained antibody responses in swine

We next evaluated the oral levamisole strategy in swine, the principal target species for FMD vaccination. The overall experimental design is summarized in Figure 3A. Oral levamisole administration in combination with intramuscular vaccination was associated with enhanced SP-specific antibody responses against both serotype O and serotype A compared with vaccination alone (Figures 3B,C). Consistent with these findings, VN titers against O PA2 and A YC were also higher in the levamisole-treated group at multiple sampling time points (Figures 3D,E). Overall, oral levamisole administration was associated with higher and more sustained SP ELISA and VN antibody responses than vaccination alone.

**Figure 3:**
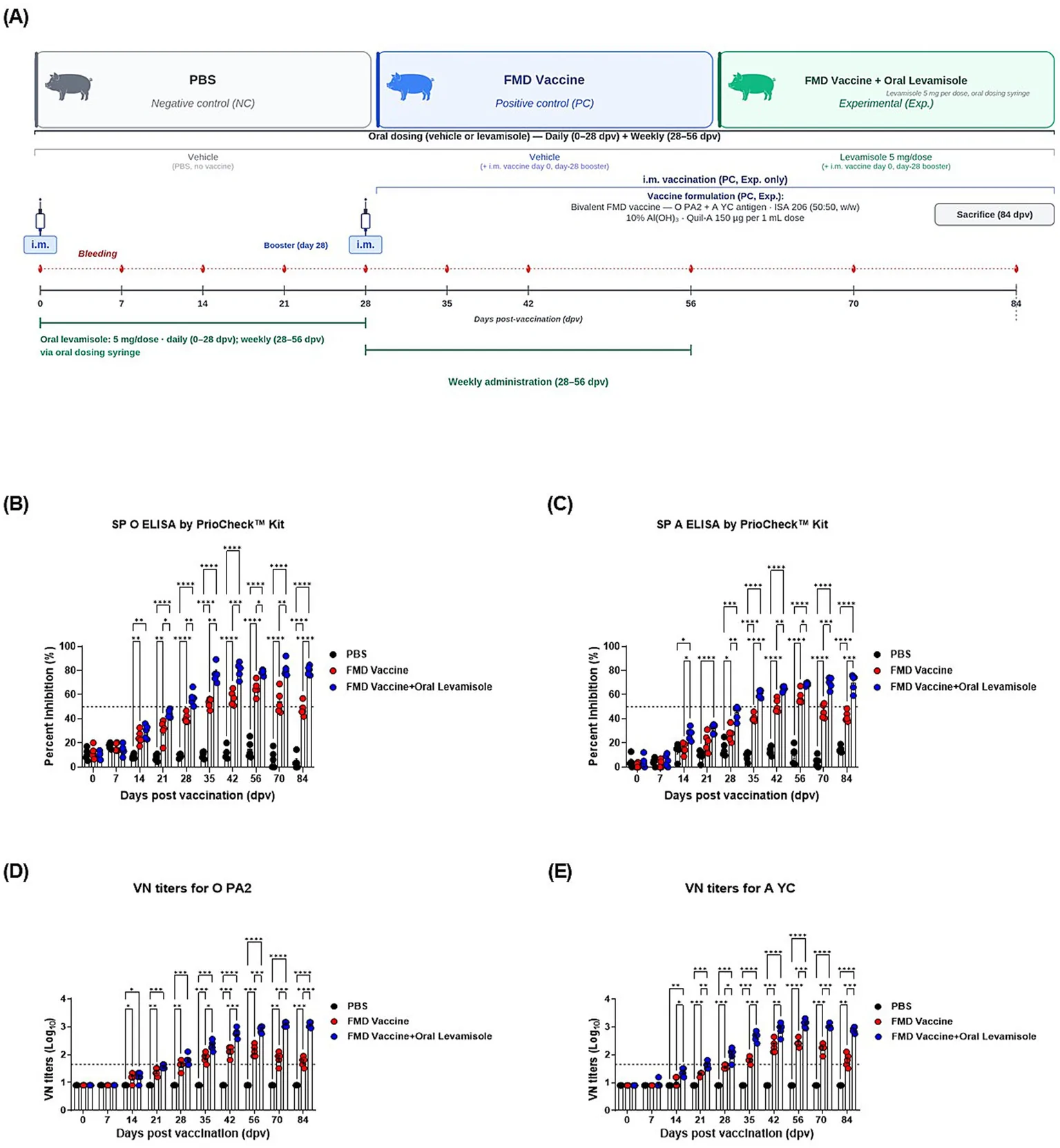
Oral levamisole administration enhanced and sustained vaccine-induced antibody responses in swine. **(A)** Schematic overview of the swine study design. Swine were immunized intramuscularly with a bivalent inactivated FMD vaccine on day 0 and received a booster vaccination on day 28, while oral levamisole was administered according to the indicated schedule. **(B,C)** SP-specific antibody responses against serotypes O and A, respectively. **(D,E)** VN antibody titers against the O PA2 and A YC strains, respectively. Oral levamisole administration was associated with stronger and more sustained humoral responses than vaccination alone throughout the observation period. Data are presented as mean ± SEM. Statistical significance is indicated as follows: *p < 0.05, **p < 0.01, ***p < 0.001, and ****p < 0.0001. Statistical significance: two-way repeated-measures ANOVA (Geisser–Greenhouse) with Tukey-adjusted *p* values.

### 3.4 Oral levamisole was well tolerated in swine

To assess the tolerability of repeated oral levamisole administration, swine were monitored clinically throughout the study and further evaluated by serum biochemical analyses. No overt treatment-related abnormalities were observed during the experimental period. Moreover, liver- and kidney-related biochemical parameters, including ALT, AST, BUN, and creatinine, remained within expected reference ranges and showed no evidence of treatment-associated toxicity (Table 1). These observations supported the tolerability of oral levamisole administration under the study conditions.

**Table 1:** Serum biochemistry parameters in swine following oral levamisole administration in combination with intramuscular FMD vaccination.

Group	Days post-vaccination (dpv)	ALT (U/L)	AST (U/L)	BUN (mg/dL)	CREA (mg/dL)	LDH (U/L)	TP (mg/dL)	ALB (mg/dL)	A/G ratio
PBS	0	41.20 ± 3.68	40.00 ± 2.06	5.90 ± 0.37	0.75 ± 0.02	421.28 ± 45.74	2.58 ± 0.10	2.90 ± 0.06	1.22 ± 0.04
	28	56.80 ± 4.03	46.33 ± 12.25	9.56 ± 1.08	1.01 ± 0.05	496.23 ± 40.97	6.08 ± 0.15	3.36 ± 0.05	1.26 ± 0.08
	56	49.60 ± 1.85	43.20 ± 13.28	10.50 ± 1.21	1.15 ± 0.02	333.10 ± 13.02	6.72 ± 0.15	3.14 ± 0.08	0.89 ± 0.05
	84	47.00 ± 1.74	46.00 ± 14.04	17.26 ± 1.47	1.46 ± 0.05	266.48 ± 2.76	6.06 ± 0.07	3.62 ± 0.04	1.49 ± 0.05
FMD Vaccine	0	37.20 ± 1.73	32.60 ± 0.61	7.50 ± 1.43	0.68 ± 0.02	340.30 ± 13.22	5.34 ± 0.07	2.98 ± 0.07	1.26 ± 0.08
	28	48.40 ± 1.85	45.50 ± 3.32	9.30 ± 0.67	0.93 ± 0.02	456.34 ± 17.62	6.30 ± 0.14	3.32 ± 0.11	1.12 ± 0.07
	56	53.80 ± 3.33	48.60 ± 9.20	13.98 ± 0.95	1.16 ± 0.05	361.90 ± 26.52	6.80 ± 0.10	3.36 ± 0.08	0.98 ± 0.02
	84	43.60 ± 2.79	47.75 ± 5.86	16.78 ± 1.60	1.30 ± 0.07	254.12 ± 8.38	6.34 ± 0.12	3.72 ± 0.09	1.44 ± 0.08
FMD Vaccine + Oral Levamisole	0	41.60 ± 3.67	37.60 ± 2.94	5.48 ± 0.28	0.78 ± 0.03	401.34 ± 28.86	5.58 ± 0.16	3.06 ± 0.13	1.22 ± 0.07
	28	55.00 ± 6.83	46.60 ± 5.62	6.40 ± 0.42	0.98 ± 0.03	525.40 ± 93.45	6.28 ± 0.14	3.20 ± 0.19	1.07 ± 0.11
	56	46.60 ± 3.61	42.40 ± 4.46	12.42 ± 2.41	1.17 ± 0.05	394.54 ± 14.78	7.02 ± 0.19	3.36 ± 0.16	0.96 ± 0.12
	84	39.00 ± 1.92	40.60 ± 2.56	15.94 ± 1.46	1.38 ± 0.06	249.90 ± 13.44	6.44 ± 0.05	3.64 ± 0.04	1.31 ± 0.05

Serum biochemical parameters, including markers of liver and kidney function, were measured at the indicated time points in swine from each experimental group. No treatment-related abnormalities were observed under the study conditions. Data are presented as mean ± SEM. Statistical analyses were performed as described in the Materials and Methods section. Reference ranges should be interpreted according to institutional guidelines or species-specific standards.

### 3.5 Oral levamisole increased IgA-associated immune responses

As FMDV infection is closely linked to mucosa-associated tissues, we next examined whether oral levamisole influenced sIgA-associated immune responses. In mice, oral levamisole administration was associated with increased serum IgA-associated responses, accompanied by a corresponding increase in salivary sIgA-associated signals (Figure 4A; Supplementary Figure 1). Similarly, increased serum IgA-associated responses were detected in levamisole-treated swine during the post-vaccination period (Figure 4B). Although these measurements do not independently establish local mucosal protection, they are consistent with activation of immune pathways associated with mucosal immune responses following oral levamisole administration.

**Figure 4:**
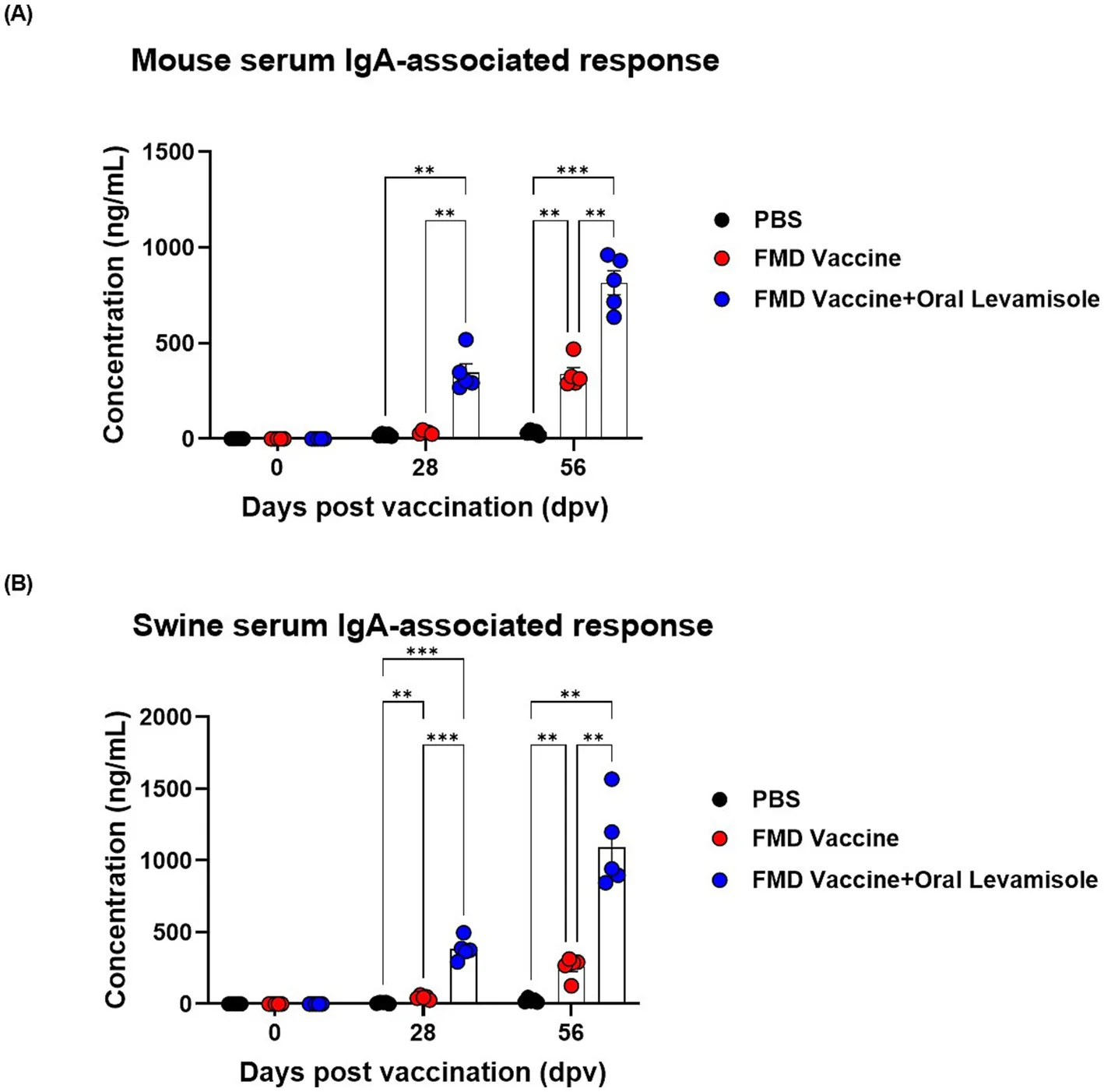
Impact of oral levamisole on IgA-associated responses. **(A)** Mouse serum IgA-associated responses. **(B)** Serum IgA-associated responses in swine. In mice, sIgA was quantified using a mouse sIgA-specific ELISA kit (CSB-E08413m; Cusabio), whereas swine serum samples were analyzed using a porcine sIgA-specific ELISA kit (Cloud-Clone Corp.). Because the swine measurement was performed in serum, the results are reported conservatively as serum IgA-associated responses and were not interpreted as direct evidence of protective mucosal immunity. Data are presented as mean ± SEM (*n* = 5 per group).

### 3.6 Oral levamisole altered immune-related cytokine gene expression in swine

To further characterize the immunological effects of oral levamisole administration, we analyzed the expression of IL-2, IL-4, IL-12p40, IL-17A, IL-18, IL-23p19, IL-23R, and IFNγ in porcine PBMCs by quantitative RT-PCR. As shown in Figure 5, oral levamisole treatment was associated with increased expression of these immune-related genes compared with vaccination alone. The upregulation of IL-12p40 and IL-18, together with increased IFNγ expression, was consistent with a Th1-associated antiviral cytokine profile. Increased IL-2 expression may have reflected enhanced helper T-cell activity during the post-vaccination period, whereas the more modest increase in IL-4 suggested a broader helper-cell environment potentially relevant to post-vaccination immune support. In addition, the concurrent upregulation of IL-17A, IL-23p19, and IL-23R was consistent with activation of immune pathways involved in barrier-site immune coordination and mucosa-associated immunity. Overall, the transcriptional profile suggested broader cytokine engagement rather than a strictly polarized Th1- or Th2-dominant response.

**Figure 5:**
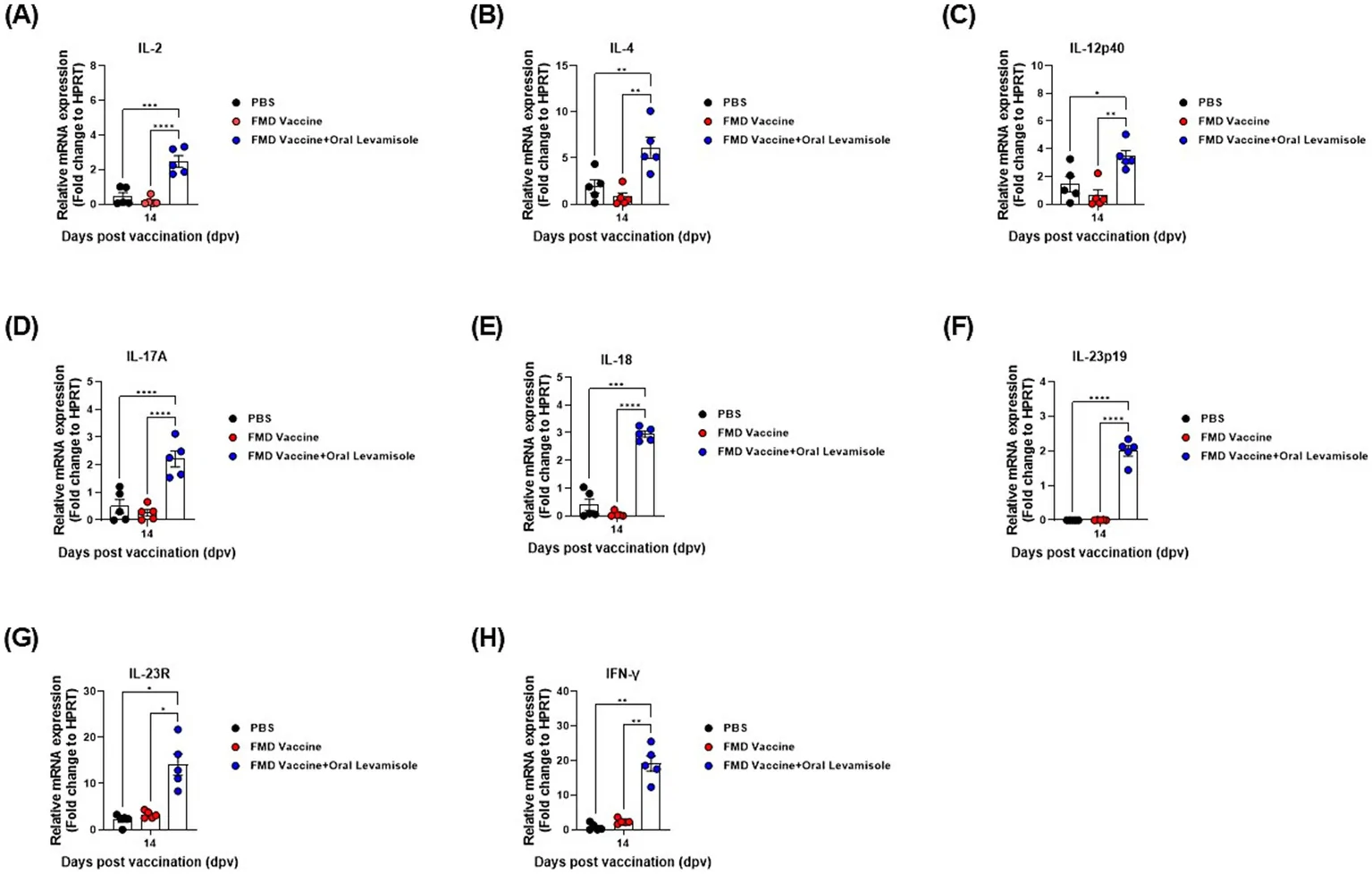
Oral levamisole administration altered immune-related cytokine gene expression in porcine PBMCs. Relative expression levels of **(A)** IL-2, **(B)** IL-4, **(C)** IL-12p40, **(D)** IL-17A, **(E)** IL-18, **(F)** IL-23p19, **(G)** IL-23R, and **(H)** IFN-γ were measured by quantitative RT-PCR in PBMCs isolated from swine at the indicated time points after vaccination. Oral levamisole treatment was associated with increased expression of these cytokine-related genes compared with vaccination alone, consistent with broader cytokine-related immune activation involving antiviral and mucosal immune pathways. Gene expression levels were normalized to HPRT and are presented as fold changes relative to the corresponding control group. Data are shown as mean ± SEM. Statistical significance is indicated as follows: *p < 0.05, **p < 0.01, ***p < 0.001, and ****p < 0.0001. Ordinary one-way ANOVA with Tukey’s test; for IFN-γ, IL-18, and IL-23R (unequal variances), Welch’s ANOVA with Dunnett’s T3 test was used.

## 4 Discussion

This study examined a practical question: whether levamisole retains immunomodulatory utility when administered independently of the vaccine formulation. Under the conditions tested, separate oral administration was associated with enhanced serological responses in both mice and swine, improved post-challenge outcomes in mice, increased IgA-associated responses, cytokine-related transcriptional changes in porcine PBMCs, and acceptable tolerability in swine.

The present study extends our previous work (22), in which levamisole was incorporated directly into the injected FMD vaccine as a formulation-integrated adjuvant. In contrast, levamisole was administered orally and independently of vaccination in the present study, separating adjunct administration from vaccine delivery in both route and dosing schedule. Despite this fundamental difference in administration mode, enhanced serotype-specific antibody and virus-neutralizing responses were observed in both mice and swine. In mice, these serological improvements were accompanied by improved survival and reduced post-challenge body weight loss following FMDV challenge. Thus, the present findings extend the previous formulation-integrated approach by showing that the immunomodulatory activity of levamisole can also be achieved through a separate oral regimen.

The swine study addressed a key question for veterinary application: whether the same strategy could enhance vaccine responsiveness in a target species. Oral levamisole administration resulted in higher and more sustained antibody responses, together with increased virus-neutralizing antibody titers across the observation period (Figures 3B–E). These findings support the relevance of the immunomodulatory effect of oral levamisole in a principal target species for FMD vaccination (17).

The complementary use of mice for challenge-based proof-of-concept assessment and swine for target-species evaluation provided biological and veterinary context for the observed effects (17, 28).

Operational viability also depends on tolerability. Repeated oral administration of levamisole did not produce overt treatment-related abnormalities, and the serum biochemistry profile showed no evidence of clinically relevant hepatic or renal toxicity (Table 1). For an adjunct intended for routine livestock use, this consideration is critical. A regimen that improves immunogenicity but introduces handling difficulties or safety concerns would have limited practical value.

Another notable observation was the increase in IgA-associated responses following oral adjunct administration. Increased signals were detected in mouse serum and saliva, as well as in swine serum, during the post-vaccination period (Figures 4A,B; Supplementary Figure 1). This study did not evaluate tissue-level mucosal immunity, local viral replication, or viral shedding; therefore, the IgA-associated findings should be interpreted cautiously. Nevertheless, the results are consistent with the possibility that separate oral immunomodulation can influence immune compartments that are not typically captured by serum antibody measurements alone, thereby complementing the conventional immunological profile generated by intramuscular FMD vaccination (12, 29).

The transcriptional profile observed in porcine PBMCs provides additional insight into potential mechanisms underlying these effects. Levamisole has been reported to modulate antigen-presenting-cell activation and maturation, lymphocyte responsiveness, and broader immune-cell signaling (20, 21). In the present study, increased expression of IL-12p40, IL-18, and IFNγ was consistent with engagement of Th1-associated antiviral pathways, whereas increased IL-2 may reflect enhanced lymphocyte activation. Concurrent changes in IL-17A, IL-23p19, and IL-23R further suggest engagement of pathways involved in barrier-associated immune coordination (Figure 5). Together with the IgA-associated responses, these findings are consistent with broad modulation of the post-vaccination immune environment rather than enhancement of a single effector pathway. However, because the present study assessed cytokine-related transcription rather than pathway-specific functional mechanisms, these interpretations require direct mechanistic validation.

From a practical veterinary perspective, independent oral administration may offer flexibility because adjunct dosing can be implemented without modifying the manufacture or composition of existing injectable FMD vaccines. This approach could potentially allow additional immune support to be provided during defined periods after vaccination or in animals with suboptimal vaccine responsiveness. Oral administration may also facilitate repeated adjunct dosing without additional vaccine injections. However, the regimen evaluated in this proof-of-concept study involved repeated administration and should not yet be considered field-ready. Practical application will require evaluation of simplified dosing schedules, efficacy under commercial production conditions, and confirmation of protection and long-term safety in target species.

Several limitations should be recognized. This study did not include a levamisole-only control group; consequently, the FMDV-specific responses reported here cannot fully distinguish enhancement of vaccine-induced immunity from any antigen-independent immunomodulatory activity of levamisole, and future studies will incorporate a levamisole-only arm. The vaccine strain O PA2 (O/PAK/44/2008) and the challenge strain O/VET/2013 are distinct isolates within serotype O; the mouse challenge therefore represents an intratypic (within-serotype O) heterologous exposure rather than a strictly homologous challenge. Serotype O was used because it has been the predominant and epidemiologically most relevant serotype in the Republic of Korea. Protective challenge data were obtained only in mice, tissue-level mucosal immune responses and protein-level cytokine measurements were not evaluated, and the observation period was limited.

Although no overt treatment-related abnormalities or clinically relevant hepatic or renal biochemical changes were detected under the present experimental conditions, these findings do not exclude subtler effects of prolonged immune modulation. The PBMC data reflect cytokine-related gene transcription and do not establish sustained systemic cytokine protein production. Nevertheless, excessive or prolonged immune activation could potentially influence inflammatory homeostasis, growth, feed efficiency, or stress responses in production settings. Longer-term field-relevant studies incorporating performance measures and broader inflammatory and safety endpoints are therefore required. Despite these limitations, the present findings support oral levamisole as a feasible formulation-preserving adjunct for enhancing intramuscular FMD vaccination.

## 5 Conclusion

Oral levamisole strengthened antibody and virus-neutralizing responses to intramuscular FMD vaccination in both mice and swine, improved post-challenge outcomes in mice, and was well tolerated under the porcine study conditions. These results support levamisole as a practical route-decoupled adjunct for enhancing vaccine performance while preserving the original injectable formulation. More broadly, the results indicate that oral levamisole provides proof-of-concept evidence for a candidate immunomodulatory strategy for improving vaccine responsiveness without requiring modification of established vaccine formulations.

## Data Availability

The original contributions presented in the study are included in the article/Supplementary material, further inquiries can be directed to the corresponding author.
