# Supplementary material for: Oral levamisole as a route-decoupled adjunct to intramuscular foot-and-mouth disease vaccination: a two-species evaluation
Source: Front Vet Sci. 2026 Sep 23;13:1918567. doi: 10.3389/fvets.2026.1918567 (PMC13643919; doi:10.3389/fvets.2026.1918567)

Supplementary Material

Oral levamisole as a route-decoupled adjunct to intramuscular foot-and-mouth disease vaccination: a two-species evaluation

Hyeong Won Kim, So Hui Park, Mi-Kyeong Ko, Seokwon Shin, Jong-Hyeon Park, Min Ja Lee*

Center for Foot-and-Mouth Disease Vaccine Research, Animal and Plant Quarantine Agency, 177 Hyeoksin 8-ro, Gimcheon-si, Gyeongsangbuk-do 39660, Republic of Korea

***Correspondence:**
Min Ja Lee
herb12@korea.kr

1 Supplementary Tables and Figures

1.1 Supplementary Tables

Supplementary Table 1. Primer sequences used for quantitative RT-PCR analysis of immune-related genes.

Target	Forward/Reverse	Sequence (5′-3′)	Length (mer)
IL-2	IL-2 F	AAGCTCTGGAGGGAGTGCTA	20
	IL-2 R	CAACAGCAGTTACTGTCTCATCA	23
IL-4	IL-4 F	CTCACCTCCCAACTGATCCC	20
	IL-4 R	TGTGTCCGTGGACGAAGTTG	20
IL-12p40	IL-12p40 F	GGAGTATAAGAAGTACAGAGTGG	23
	IL-12p40 R	GATGTCCCTGATGAAGAAGC	20
IL-17A	IL-17A F	CTCGTGAAGGCGGGAATCAT	20
	IL-17A R	GGTGTGCTCCGGTTCAAGAT	20
IL-18	IL-18 F	AGCTGAAAACGATGAAGACCTG	22
	IL-18 R	AAACACGGCTTGATGTCCCT	20
IL-23p19	IL-23p19 F	CCATATCCAGTGCGGGGATG	20
	IL-23p19 R	AGGCCTTGGTGGATCCTTTG	20
IL-23R	IL-23R F	TCCCTCATTGCAAAGCACAA	20
	IL-23R R	GCATCTCCTCTTGCAAGCAAAT	22
IFNγ	IFN-γ F	GCCATTCAAAGGAGCATGGAT	21
	IFN-γ R	CTGATGGCTTTGCGCTGGAT	20
HPRT	HPRT F	CCCAGCGTCGTGATTAGTGA	20
	HPRT R	GCCGTTCAGTCCTGTCCATA	20

Forward and reverse primer sequences used for quantitative RT-PCR analysis are listed for each target gene. Gene symbols and amplicon information are presented according to the assay design used in this study.

Supplementary Table 2. Body weights of mice following oral levamisole administration during the post-vaccination period.

Group	0 dpv	7 dpv	14 dpv	21 dpv	28 dpv	56 dpv
**NC**	18.68±0.56	19.93±0.56	20.85±0.85	21.39±0.86	21.54±0.62	23.13±1.19
**PC**	19.10±0.68	20.84±0.68	21.00±0.98	21.57±0.78	21.70±0.79	24.04±0.62
**Exp**	19.39±0.34	21.24±0.45	21.26±0.56	22.28±0.42	22.34±0.57	24.92±0.46

C57BL/6 mice received oral levamisole in the experimental (Exp) group, whereas the positive control (PC) group received vaccination without levamisole. Mice in the Exp and PC groups were vaccinated intramuscularly with the FMD vaccine. Data are presented as mean ± SEM (n = 5 per group). Body-weight trajectories were analyzed by two-way repeated-measures ANOVA (Geisser–Greenhouse correction) followed by Tukey's multiple-comparison test.

dpv, days post-vaccination; NC, negative control; PC, vaccine-only positive control; Exp, vaccine-plus-levamisole group.

Supplementary Table 3. Body weight gain, food intake, and food efficiency ratio (FER) of mice following oral levamisole administration during the post-vaccination period.

Group	Weight gain (g/56 dpv)	Food intake (g/56 dpv)	FER
**NC**	4.45±1.37	121.57±3.45	3.66±1.12
**PC**	4.85±1.15	126.03±2.68	3.84±0.56
**Exp**	4.39±1.12	117.66±2.24	3.73±0.43

C57BL/6 mice received oral levamisole in the experimental (Exp) group, whereas the positive control (PC) group received vaccination without levamisole. Mice in the Exp and PC groups were vaccinated intramuscularly with the FMD vaccine. FER was calculated as body weight gain (g/56 dpv) divided by food intake (g/56 dpv) × 100. Data are presented as mean ± SEM (n = 5 per group). Body-weight gain, food intake, and FER were analyzed separately by ordinary one-way ANOVA followed by Tukey's multiple-comparison test.

dpv, days post-vaccination; FER, food efficiency ratio; NC, negative control; PC, vaccine-only positive control; Exp, vaccine-plus-levamisole group.

Supplementary Table 4. Summary of statistical analyses.

Longitudinal endpoints (Figures 1, 3, and 4) were analyzed by two-way repeated-measures ANOVA with the Geisser–Greenhouse correction (no missing values in any dataset; n = 15 animals per endpoint), followed by Tukey's multiple-comparison test with multiplicity-adjusted p values within each time point. Virus-neutralizing titers were log10-transformed before analysis. Mouse salivary sIgA (single time point, 56 dpv) was analyzed by ordinary

one-way ANOVA followed by Tukey's test. Swine PBMC cytokine gene transcription (Figure 5; single time point, 14 dpv) was analyzed by ordinary one-way ANOVA followed by Tukey's test; for IFN-γ, IL-18, and IL-23R , which showed evidence of unequal variance, Welch's ANOVA followed by Dunnett's T3 test was used. Survival (Figure 2A) was analyzed by the Kaplan–Meier method with the log-rank (Mantel–Cox) test. Analyses were performed using GraphPad Prism (version 10.2.3).

**Endpoint**	**Figure**	**Analysis**	**Treatment effect**
Mouse SP-O ELISA	Fig. 1B	Two-way RM ANOVA (GG)	$F(3.792, 22.75) = 28.26$, $p < 0.0001$
Mouse SP-A ELISA	Fig. 1C	Two-way RM ANOVA (GG)	$F(5.517, 33.10) = 32.82$, $p < 0.0001$
Mouse VN titer, O PA2	Fig. 1D	Two-way RM ANOVA (GG), $\log_{10}$	$F(2, 12) = 770.5$, $p < 0.0001$
Mouse VN titer, A YC	Fig. 1E	Two-way RM ANOVA (GG), $\log_{10}$	$F(2, 12) = 1851$, $p < 0.0001$
Mouse serum IgA-associated responses	Fig. 4A	Two-way RM ANOVA (GG)	$F(3.277, 19.66) = 66.68$, $p < 0.0001$
Mouse saliva sIgA (56 dpv)	Suppl. Fig. S1	One-way ANOVA + Tukey	$F(2, 12) = 60.53$, $p < 0.0001$; Tukey: PBS vs V ns; PBS vs V+LEV, V vs V+LEV $p < 0.0001$
Pig SP-O ELISA	Fig. 3B	Two-way RM ANOVA (GG)	$F(8.775, 52.65) = 56.43$, $p < 0.0001$
Pig SP-A ELISA	Fig. 3C	Two-way RM ANOVA (GG)	$F(8.338, 50.03) = 40.01$, $p < 0.0001$
Pig VN titer, O PA2	Fig. 3D	Two-way RM ANOVA (GG), $\log_{10}$	$F(2, 12) = 363.0$, $p < 0.0001$
Pig VN titer, A YC	Fig. 3E	Two-way RM ANOVA (GG), $\log_{10}$	$F(2, 12) = 1210$, $p < 0.0001$
Swine serum IgA	Fig. 4B	Two-way RM ANOVA (GG)	$F(2.172, 13.03) = 35.96$, $p < 0.0001$
Mouse survival (O/VET/2013 challenge)	Fig. 2A	Kaplan–Meier, log-rank (Mantel–Cox)	$\chi^2 = 11.04$, df = 2, $p = 0.0040$; survivors: PBS 0/5 (95% CI, 0–45.1%), vaccine 2/5 (95% CI, 5.3–85.3%), vaccine + levamisole 5/5 (95% CI, 47.8–100%)
IFN-γ (PBMC, 14 dpv)	Fig. 5H	Welch's ANOVA + Dunnett's T3	$W(2, 6.99) = 31.63$, $p = 0.0003$
IL-18 (PBMC, 14 dpv)	Fig. 5E	Welch's ANOVA + Dunnett's T3	$W(2, 6.20) = 285.1$, $p < 0.0001$

IL-23R (PBMC, 14 dpv)	Fig. 5G	Welch's ANOVA + Dunnett's T3	W (2, 6.78) = 11.80, $p = 0.0062$

Full model statistics (all effects, degrees of freedom, and F values) and the complete set of Tukey/Dunnett T3-adjusted pairwise comparisons with 95% confidence intervals are provided in the accompanying Supplementary Statistical Analysis workbook (sheets ordered by figure: Figure 1_Mouse, Figure 2_Survival, Figure 3_Pig, Figure 4_IgA, Figure 5_Cytokines).

1.2 Supplementary Figures

Supplementary Figure 1. Salivary Secretory IgA (sIgA)-associated responses in mice following oral levamisole administration. Saliva samples were collected from mice after pilocarpine hydrochloride administration and analyzed for sIgA-associated responses by ELISA. Data are presented as mean ± SEM, with statistical comparisons performed as described in the Methods section of the main manuscript. sIgA was quantified using a mouse secretory IgA-specific ELISA kit (CSB-E08413m; Cusabio, Wuhan, China).

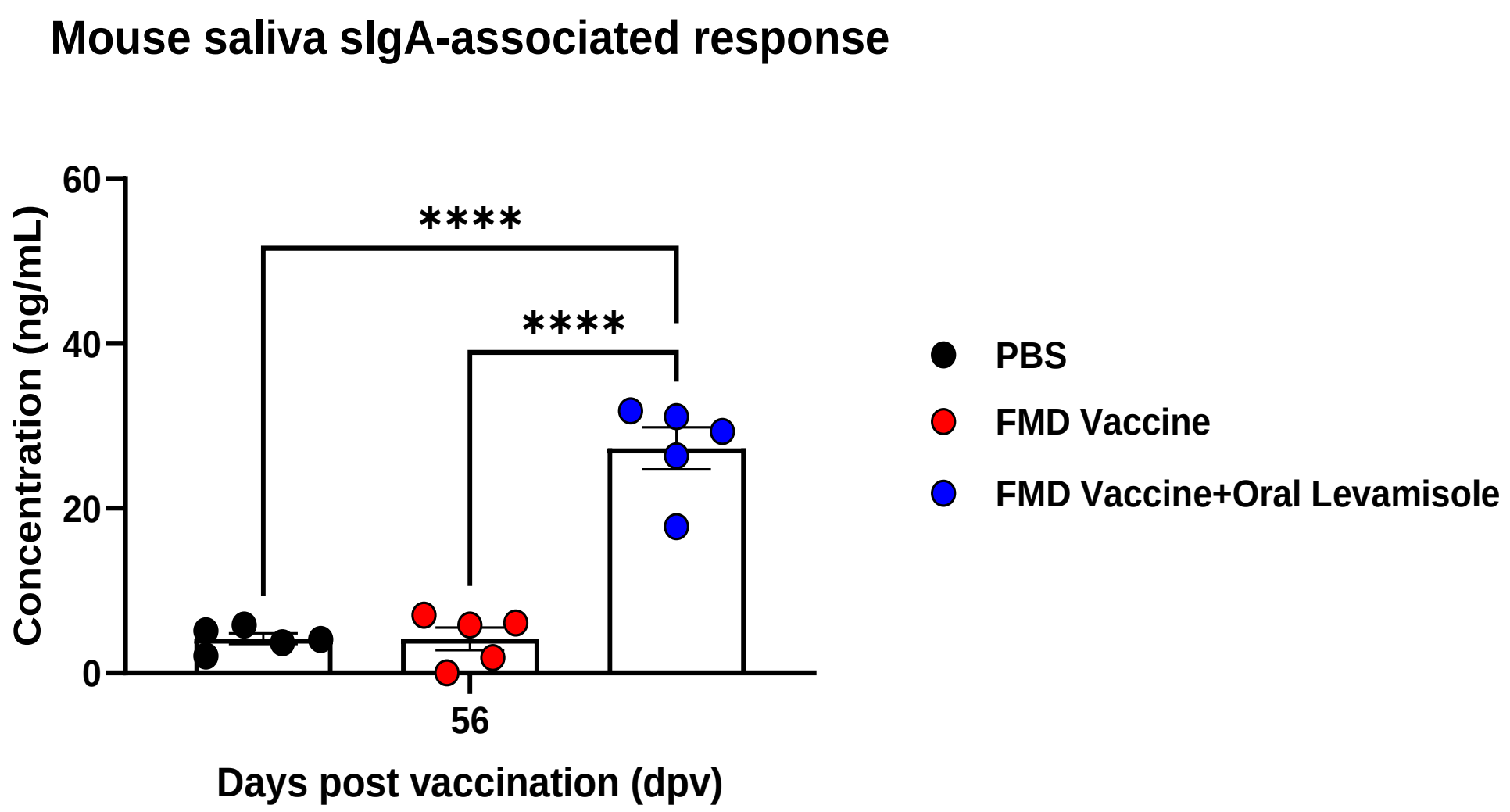

Supplement: Supplementary file 1 [file Data_Sheet_1.pdf]
